# Supplementary material for: Large-Area Fabrication of Structurally Colored and Humidity Sensitive Composite Nanofilm via Ultrasonic Spray-Coating
Source: Polymers (Basel). 2021 Oct 30;13(21):3768. doi: 10.3390/polym13213768 (PMC8587582; doi:10.3390/polym13213768)
Supplement: Supplementary file 1 [file polymers-13-03768-s001.zip › Supplementary Information-Wei Ma.pdf]

Supplementary Information

Large-area fabrication of structurally colored and humidity sensitive SiO_2 nanofilm via ultrasonic spray-coating

Sijun Li, Donghui Kou, Shufen Zhang, and Wei Ma*

State Key Laboratory of Fine Chemicals, Dalian University of Technology, Dalian, Liaoning 116023, P. R. China.

E-mail: weima@dlut.edu.cn; Tel: +86-411-84986265.

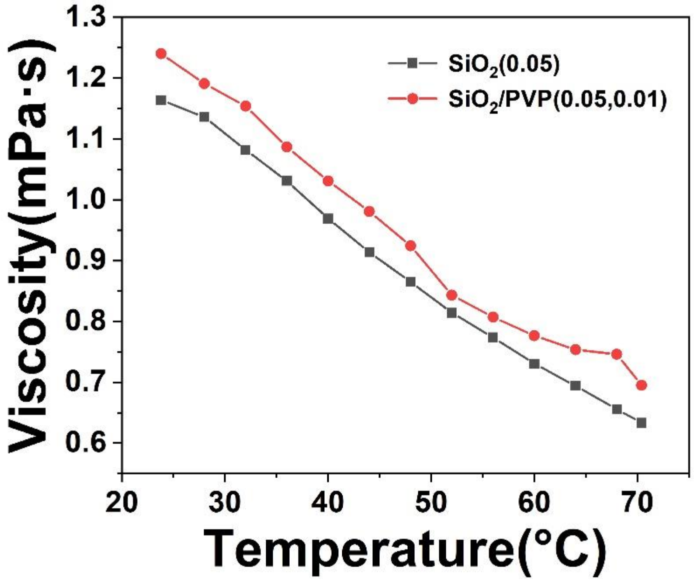

Figure S1. Viscosity of the spray solutions at different temperatures.

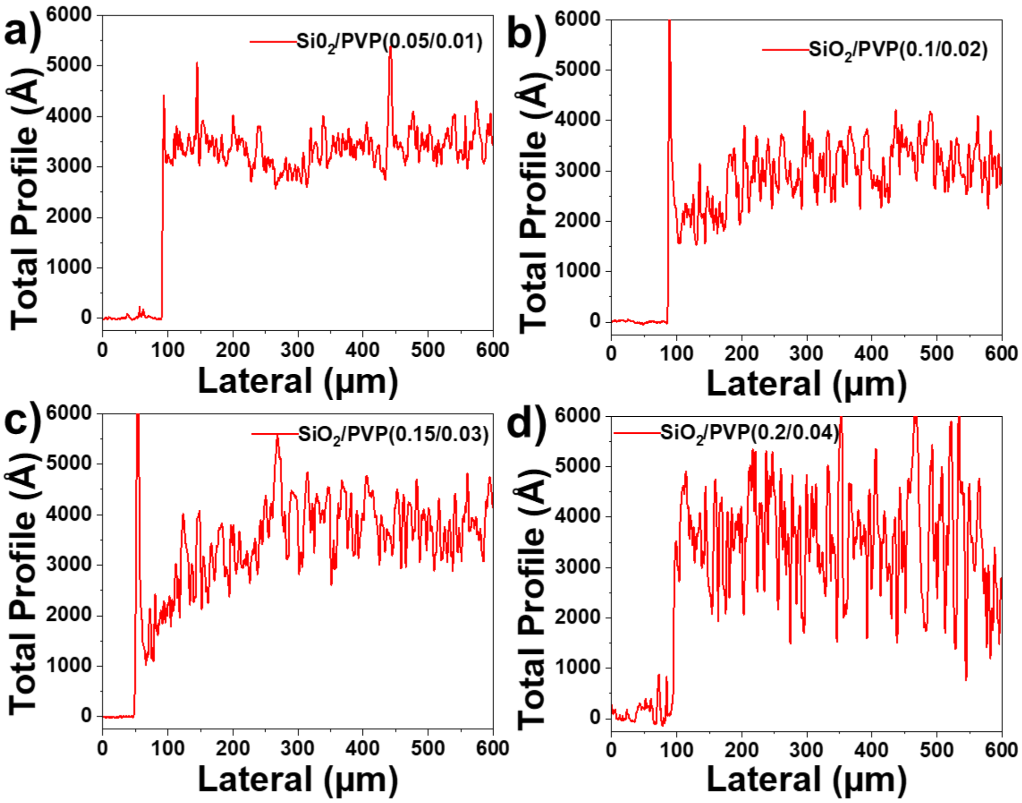

Figure S2. Film surface profile of spraying at (**a**) SiO_2/PVP (0.05/0.01), (**b**) SiO_2/PVP (0.10/0.02), (**c**) SiO_2/PVP (0.15/0.03) and (d) SiO_2/PVP (0.20/0.04) at T = 50 °C.

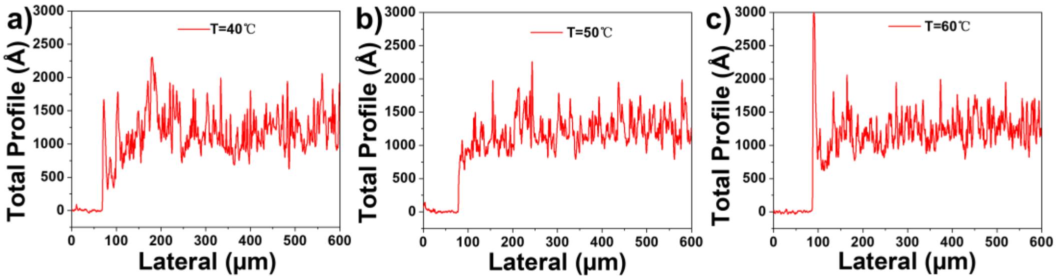

Figure S3. Film surface profile at (**a**) T=40 °C, (**b**) 50 °C and (**c**) 60 °C at SiO_2/PVP (0.05/0.01) and H=60 mm.

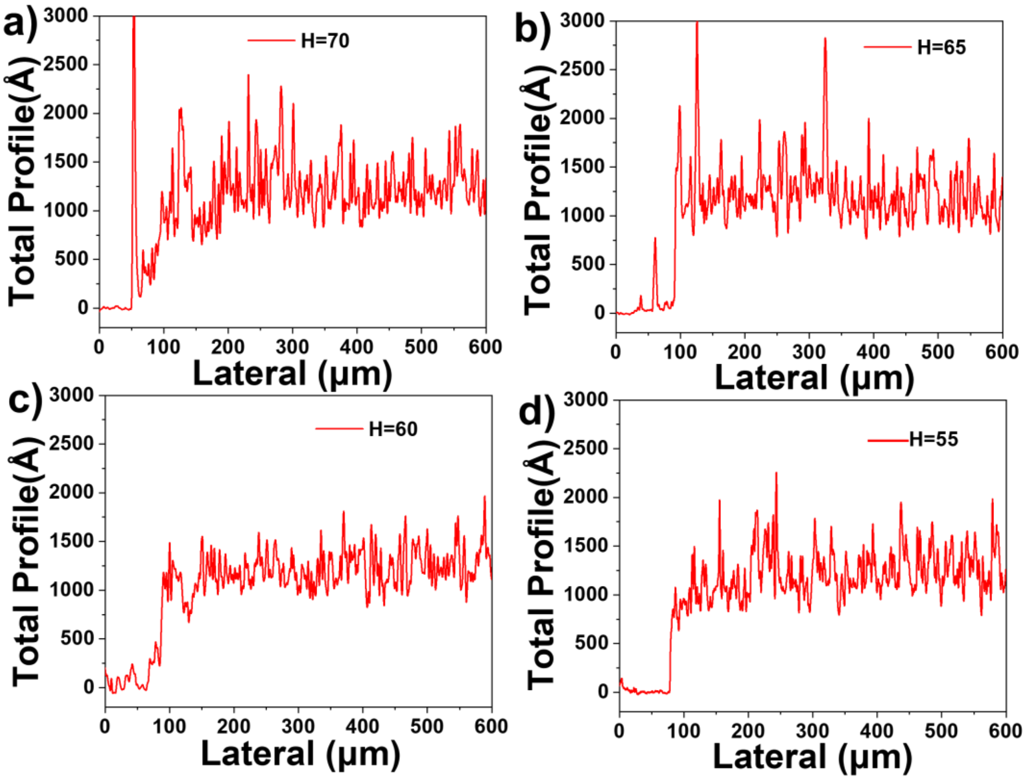

Figure S4. Film surface profile at (**a**) H = 70, (**b**) H = 65, (**c**) H = 60 and (**d**) H = 55 mm, at SiO_2/PVP (0.05/0.01) and T = 50 °C.

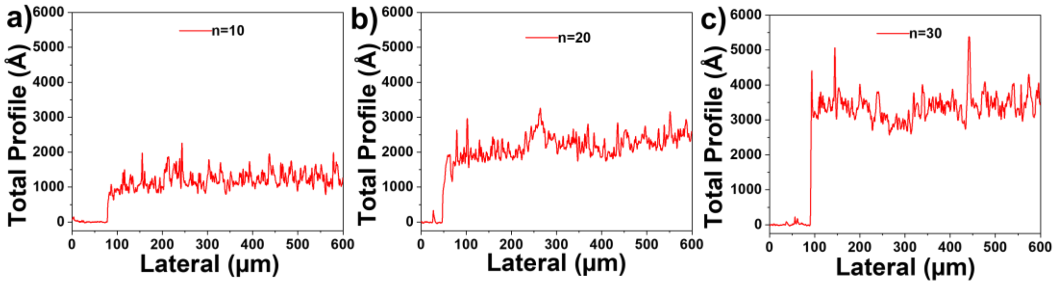

Figure S5. Film surface profile of spray passes at (**a**) N = 10, (**b**) N = 20, and (**c**) N = 30, at SiO_2/PVP (0.05/0.01), T=50 °C and H = 60 mm.

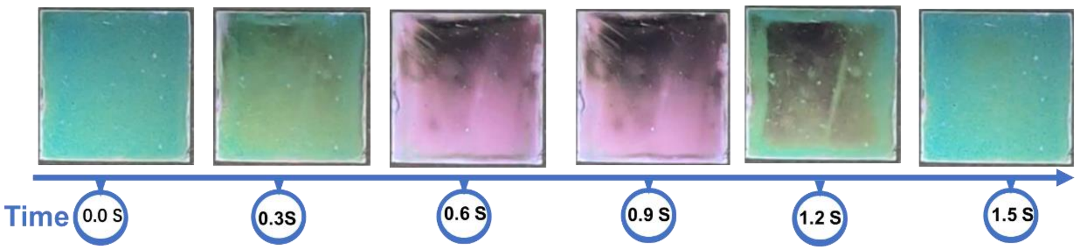

Figure S6. The response and recovery time of humidity sensing.

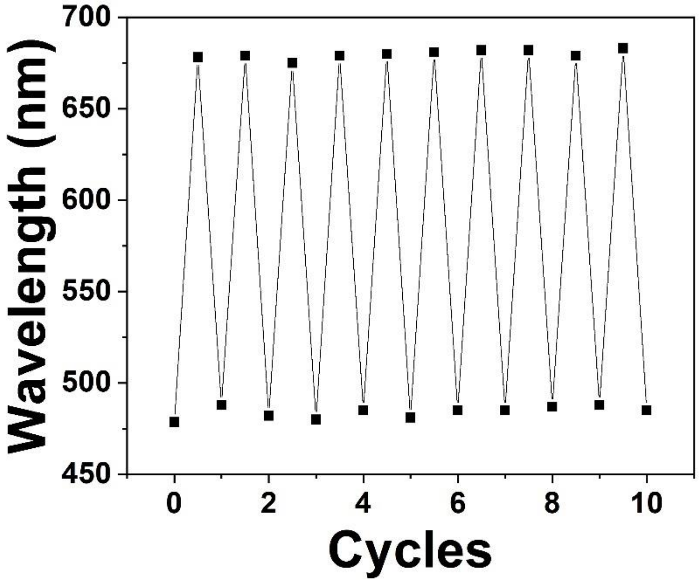

Figure S7. Recycle detection test of the structure color film in 33 % and 97 % relative humidity environment.

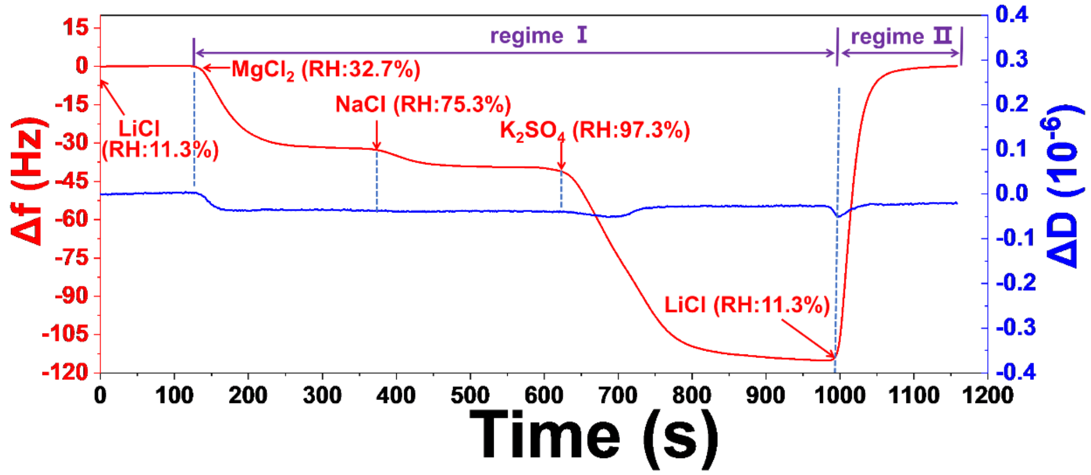

Figure S8. Variations of frequency and dissipation by spin-coating SiO_2/PVP on a gold-coated chip when exposed to different relative humidity conditions.

Table S1. Surface tension of the solutions.

Solution	Surface tension (mN / m)
SiO_2 (0.05)	71.7
SiO_2/PVP (0.05/0.01)	68.8

Table S2. Structure color film thickness.

Spray pass	Thickness (nm)
20	225.6
22	248.3
24	270.1
26	295.4
28	313.4
30	337.2
